# Hemispheric asymmetries in resting-state connectivity: insights from healthy controls and implications for neurological disorders

**DOI:** 10.1007/s00429-025-03039-8

**Published:** 2025-11-10

**Authors:** Gergo Bolla, Ahee Lee, Dalida Borbala Berente, Orsolya Szalmas, Tunde Mangel, Anita Kamondi, Andras Attila Horvath

**Affiliations:** 1 School of PhD Studies, Semmelweis University, 57 Lehel ut 1135 Budapest, Hungary; 2 Faculty of Medicine, Semmelweis University, Budapest, Hungary; 3 Neurocognitive Research Centre, Nyírő Gyula National Institute of Psychiatry and Addictology, Budapest, Hungary; 4 Department of Neurosurgery and Neurointervention, Semmelweis University, Budapest, Hungary; 5 Department of Neurology, Semmelweis University, Budapest, Hungary; 6 Department of Anatomy Histology and Embryology, Semmelweis University, Budapest, Hungary; 7 Research Centre for Natural Sciences, HUN-REN, Budapest, Hungary; 8 Universidade de Lisboa, Faculty of Medicine, Lisbon, Portugal

**Keywords:** Brain lateralisation, Resting-state fMRI, Functional connectivity, Hemispheric asymmetry, Graph-theoretical analysis, Voxel-based metrics, Laterality index

## Abstract

The human brain exhibits intricate hemispheric asymmetries at both structural and functional levels. Recent research highlights the significance of these asymmetries in various neurological diseases, with left-sided pathologies often associated with worse clinical outcomes. This study aims to unravel the lateralisation of resting-state network connectivity in the human brain using rs-fMRI, exploring its implications for the pathophysiology of neurological disorders. In this study, we utilized a multifaceted analytical approach with seven graph-theoretical measures, three voxel-based metrics, and the laterality index. The study was conducted on two independent datasets: a Local cohort comprising 102 right-handed healthy control participants, and a comparative cohort of 86 right-handed healthy control participants from the ADNI database.Our results reveal significant lateralisation of resting-state functional network connectivity in several brain regions across both datasets. The following regions exhibited consistent significant left-sided lateralisation in both cohorts, even when using different metrics: Cerebellum (10, Crus2, 6), Frontal (Orbital, Inferior Triangular, Superior), Supramarginal (posterior), Lateral Occipital, Inferior Temporal (posterior). Right lateralisation: Cerebellum 7b, Frontal Pole, Supracalcarine, Juxtapositional Lobule. The consistency of lateralisation patterns across multiple analytical methods and independent cohorts underscores the importance of our findings. The observed lateralisation phenomena might have important implications for the pathophysiology of various neurological diseases, potentially offering insights into disease progression and prognosis.

**Supplementary Information:**

The online version contains supplementary material available at 10.1007/s00429-025-03039-8.

## Introduction

The human brain proves its true nature through intricate patterns of structural and functional hemispheric asymmetry. Hemispheric laterality refers to the specialization of the left and right brain hemispheres for distinct cognitive, sensory, and motor functions, a principle now well-characterized by large-scale neuroimaging studies. Recent research using high-quality structural and functional MRI in thousands of individuals has revealed that laterality is not a static trait but exhibits dynamic fluctuations across brain networks, with these dynamics closely linked to cognitive performance and flexibility (Wu et al. 2022). For example, while language processing remains robustly left-lateralised, attention and visuospatial functions show rightward dominance patterns confirmed in both healthy and clinical populations (Bartolomeo and Seidel Malkinson 2019; Dai et al. 2024). This functional specialization is supported by a large-scale structural connectivity analyses, which found the left hemisphere to be more efficient in regions supporting language and fine motor actions, whereas the right excels in network efficiency for areas involved in memory and visuospatial attention (Caeyenberghs and Leemans 2014; Andrulyte et al. 2024). Importantly, cross-hemispheric communication and learning-dependent interactions, rather than fixed anatomical asymmetries alone, play a crucial role in shaping these lateralised functions (Ocklenburg and Guo 2024).

Increasing evidence also highlights the significance of brain asymmetry in the context of various neurological disorders. Studies have demonstrated that grey matter atrophy in neurodegenerative disorders occurs earlier and progresses faster in the left hemisphere (Lubben et al. 2021). Epilepsy studies found that the left hemisphere shows a higher tendency for epileptogenesis, and left-sided epileptic activity is associated with poorer prognosis (Holmes et al. 2001; Varoglu 2024; Clemens et al. 2024). Investigations into the anatomical occurrence of gliomas also revealed important lateralisation aspects, such as a worse prognosis in patients with left-sided frontotemporal tumors (Cini et al. 2024). This area of research holds promise for elucidating the underlying mechanisms of disease pathogenesis and progression, with implications for personalized medicine, including the identification of early diagnostic markers, prognostic markers, and targeted interventions. However, key questions are still purely understood, for instance: why is left-sided pathology associated with worse clinical prognosis, and how do asymmetries intersect with pathogenesis and disease progression? A potential first step to understand the possible mechanisms behind is to characterize the healthy lateralisation pattern.

To answer these questions, a crucial step forward lies in elucidating structural and functional connectivity asymmetries in the brains of healthy individuals, providing a framework for understanding lateralised pathology. The methods employed in brain lateralisation studies can significantly impact the analysis results. Studies on structural cortical asymmetry have consistently found greater cortical thickness in the left frontotemporal regions compared to the right side, and in the right posterior cortical areas compared to the left side (Toga and Thompson 2003). A structural MRI study involving 15,847 subjects found that the extent of hemispheric asymmetry does not vary with age except for the volume of the putamen (Guadalupe et al. 2017). Interestingly the study found no significant effects of handedness on the asymmetry of subcortical structures, highlighting the complexity of factors that contribute to brain lateralisation including functional differences. Histopathology studies on the functional asymmetries revealed an increase in the neurite density in the left frontal, temporal, and inferior parietal cortices compared to the similar regions of the right hemisphere (Schmitz et al. 2019). Higher neurite density on the left side in these regions was also shown by Kong et al. 2020. A similar asymmetry in the arcuate fasciculus and the subinsular white matter tracts was also demonstrated in diffusion tensor imaging (DTI) studies (Rodrigo et al. 2007). Resting-state fMRI studies on network lateralisation using graph analytical methods and Voxel Mirrored Homotopic Connectivity (VMHC) showed left-sided lateralisation for auditory and default mode networks, and right-sided lateralisation for memory, visuospatial areas, and the visual networks (Zuo et al. 2010; Caeyenberghs and Leemans 2014; Agcaoglu et al. 2015). While studies on the functional lateralisation of neural connectivity might reveal an insight on the potential mechanisms behind disease related lateralisation, functional neuroimaging studies on lateralisation pattern using resting state measurements with the combination of multiple analysis techniques are missing (Lubben et al. 2021).

In this study, we present the results of a multimodal connectivity analysis performed on two independent fMRI datasets to investigate the lateralisation of the resting state network connectivity of the healthy human brain. Our approach incorporates seven graph-theoretical measures, three voxel-based metrics, and calculating the laterality index. We intend to uncover the complex neural correlates of lateralisation through these diverse techniques, focusing on the overlaps of the two independent study populations. With the proposal of a novel interpretation, we aim to explain the background of laterality preponderance which can be a potential factor in various neurological diseases.

## Materials and methods

### Datasets

#### Local cohort

For the Local dataset, we included 102 right-handed healthy control participants from the AlzEpi Cohort Observational Library, a self-developed database that focuses on distinguishing between physiological and pathological aging. Participants were recruited at the Neurocognitive Research Centre (NRC) of the Nyírő Gyula National Institute of Psychiatry and Addictology, Budapest, Hungary. All subjects were native Hungarians. Every participant underwent an extensive investigation protocol, which included a neurological examination, blood chemistry testing, a neuropsychological evaluation, and a cranial MRI. Handedness was measured with drawing and writing samples, consequently comparing the left- and right-handed characteristics by a trained neuropsychologist, and was reinforced by the Edinburgh Handedness Inventory. All participants were classified as healthy control individuals with negative neurological status, low risk of anxiety or mood disorders, and normal neuropsychological performance (Mini-Mental Score Examination > 25; Total Score of Addenbrooke Cognitive Examination > 85; Beck Depression Inventory-II Score < 13; Spielberger-Trait Anxiety Score < 45) (Horváth et al. 2016; Hoops et al. 2009). All participants had normal brain MRIs without lesions. Individuals with known risk factors of neurological disorders or a medical history of predisposing conditions were omitted. These factors were the following: vitamin B12 deficiency, hypothyroidism, liver disease, renal insufficiency, demyelinating conditions, hydrocephalus, head injury accompanied by loss of consciousness, use of psychoactive drugs, epilepsy, alcohol or substance abuse, schizophrenia, major depression, anxiety disorders, electroconvulsive therapy, HIV infection, syphilis or preceding central nervous system infections. All research activities occurred according to the applicable regulations and guidelines. All participants provided written informed consent. The Hungarian Medical Research Council authorized our research (reference number: IV/5831 3/2021/EKU).

#### Alzheimer’s Disease Neuroimaging Initiative (ADNI) cohort

Data from 86 right-handed healthy control participants were used from the ADNI database (adni.loni.usc.edu). The ADNI database includes data on 884 healthy participants. A random automated selection approach was applied, matching our local dataset in terms of sociodemographic parameters (age, sex, and education) and the neuropsychological profile (average MMSE). Finally, a group of 86 random healthy participants matching our sample was generated for comparative analysis.

### MRI data acquisition

All local cohort subjects underwent brain MRI, producing a high-resolution anatomical image that was used for further processing and analysis. A Siemens Magnetom Verio 3 T scanner (Siemens Healthcare, Erlangen, Germany) was used, along with the standard 12-channel head receiver coil. The protocol consisted of structural and functional measurements. First a T1-weighted 3D MPRAGE (magnetization prepared rapid gradient echo) anatomical imaging (TR (time resolution) = 2.300 ms; TE (echo time) = 3.4 ms; TI = 100 ms; Flip Angle: 12°; Voxel Size: 1.0 × 1.0 × 1.0 mm) was acquired, followed by a resting-state functional MRI (rs-fMRI), an EPI-based MRI sequence (TR = 2000 ms; TE = 30 ms; Flip Angle = 90°; Voxel Size = 3 × 3 × 3 mm, Matrix = 64 × 64 pixels, Pixel spacing = 3.7 mm, Slice thickness = 3 mm). The fMRI scanning lasted 10 min while the patients were lying on the table with their eyes closed. A T2-, diffusion-, and a FLAIR-weighted sequence to identify the possible pathological lesions were also performed.

The ADNI dataset comprises multiple MRI scans with very similar protocols, which were used for the examination of the local cohort. The structural MRI (sMRI) scans were the same for all ADNI subjects: 256 × 256 × 170 voxels of 1 × 1 × 1 mm^3^. Resting-state fMRI scans were performed on a 3 T Philips scanner with the following parameters: Field Strength = 3 T; Flip Angle = 80.0°; Matrix = 64 × 64 pixels; Pixel Spacing = 3.3 mm; Slice Thickness = 3.3 mm; TE = 30.0 ms; TR = 3000.0 ms. For the 3 T Siemens scanner, the scan parameters are: Field Strength = 3 T; Flip Angle = 90°; Matrix = 448 × 448 pixels; Pixel Spacing = 3.4 mm; Slice Thickness = 3.4 mm; TE = 30.0 ms; TR = 3000.0 ms. For the 3 T GE scanners, the image characteristics are: Field Strength = 3.0 T; Flip Angle = 90°; Matrix = 64 × 64 pixels; Pixel Spacing = 3.3 mm; Slice Thickness = 3.3 mm; TE = 30.0 ms; TR = 2925.0 m.

### MRI data analysis

For rs-fMRI data analysis, we used the CONN toolbox (https://web.conn-toolbox.org/) (Nieto-Castanon 2020). CONN is a MATLAB-based software that provides a comprehensive platform for the preprocessing, analysis, and visualization of rs-fMRI data. The CONN toolbox provides an automated, detailed account of rs-fMRI data processing methods, intended for verbatim inclusion in research manuscripts to promote replicability and methodological clarity in neuroimaging studies. Below is the generated text for our processing pipeline.

### fMRI image preprocessing

Preprocessing: Functional and anatomical data were pre-processed using a flexible preprocessing pipeline (Nieto-Castanon 2020) including realignment with correction of susceptibility distortion interactions, slice timing correction, outlier detection, direct segmentation, and Montreal Neurological Institute template’s space (MNI-space) normalization, and smoothing. Functional data were realigned using Statistical Parametric Mapping (SPM) realign & unwarp procedure, where all scans were co-registered to a reference image (first scan of the first session) using a least squares approach and a 6 parameter (rigid body) transformation (‘The Slice-Timing Problem in Event-Related fMRI’, n.d.), and resampled using b-spline interpolation to correct for motion and magnetic susceptibility interactions. Temporal misalignment between different slices of the functional data (acquired in interleaved Siemens order) was corrected following SPM slice-timing correction (STC) procedure (Sladky et al. 2011), using since temporal interpolation to resample each slice Blood Oxygen Level Dependent (BOLD) timeseries to a common mid-acquisition time. Potential outlier scans were identified using ART (Morfini et al. 2023) as acquisitions with framewise displacement above 0.9 mm or global BOLD signal changes exceeding 5 standard deviations (Power et al. 2014). A reference BOLD image was computed for each subject by averaging all scans excluding outliers. Functional and anatomical data were normalized into standard MNI space, segmented into grey matter, white matter, and CSF tissue classes, and resampled to 2 mm isotropic voxels following a direct normalization procedure (Smith 2001) using SPM unified segmentation and normalization algorithm (Smith 2001) with the default IXI-549 tissue probability map template. Last, functional data were smoothed using spatial convolution with a Gaussian kernel of 8 mm full width half maximum (FWHM).

Denoising: In addition, functional data were denoised using a standard denoising pipeline including the regression of potential confounding effects characterized by white matter timeseries (5 CompCor noise components), CSF timeseries (5 CompCor noise components), motion parameters and their first order derivatives (12 factors) outlier scans (below 35 factors), session and task effects and their first order derivatives (6 factors), and linear trends (2 factors) within each functional run, followed by bandpass frequency filtering of the BOLD timeseries between 0.008 Hz and 0.09 Hz. CompCor noise components within white matter and CSF were estimated by computing the average BOLD signal as well as the most significant principal components orthogonal to the BOLD average, motion parameters, and outlier scans within each subject’s eroded segmentation masks. From the number of noise terms included in this denoising strategy, the effective degrees of freedom of the BOLD signal after denoising were estimated to range from 83.6 to 171.5 (average 140.3) across all subjects (Nieto-Castanon 2020).

### Resting state-fMRI graph metrics

Seven graph-based parameters were calculated from rs-fMRI data using the CONN Toolbox: Global Efficiency (GE), Local Efficiency (LE), Betweenness Centrality (BC), Average Path Length (APL), Clustering Coefficient (CC), degree, and cost. GE is used to measure information transfer efficiency in the entire brain network. It provides insights into how effectively information is exchanged across the whole brain. It is calculated as the average of inverse distances between a node and all other nodes in the same graph (Nieto-Castanon 2020). LE measures local integration or coherence, indicating how well each subgraph exchanges information. It’s defined as the GE of the neighbouring sub-graph of each node (Nieto-Castanon 2020). BC is a measure of node centrality within a graph. BC is the proportion of times that a node is part of the shortest path between any two pairs of nodes within a graph (Nieto-Castanon 2020). APL is defined between each pair of nodes, and it’s the minimum number of edges traversed in an optimal path between them. It represents a measure of node centrality within a network and is also a measure of the interconnectedness or radius of the entire network. It’s calculated as the average path distance between a node and all other nodes in the subgraph of connected nodes (Nieto-Castanon 2020). CC is a measure of the degree to which nodes in a graph tend to cluster together. It’s defined as the proportion of connected edges in the local neighbouring sub-graph for each node. *Degree* and *cost* are related measures. The degree is the number at each node, while the cost is the proportion of edges from/to/each node (Nieto-Castanon 2020).

These metrics have been used to assess different neuropsychiatric conditions with fMRI data (Sanz-Arigita et al. 2010; de Vos et al. 2018; Khazaee et al. 2017). The same 132 ROIs were used as stated in the section on voxel-based metrics.

### Resting state-fMRI voxel-based metrics

We employed three voxel-based metrics from the CONN Toolbox: Intrinsic Connectivity (IC), Local Correlation (LCOR), and Fractional Amplitude of Low-Frequency Fluctuations (fALFF). These measures and their similar variants have been previously utilized in various neuropsychiatric conditions (Cha et al. 2015; Li et al. 2021; Zou et al. 2008; Hou et al. 2014). IC maps characterizing network centrality at each voxel were estimated as the root mean square (RMS) of all short- and long-range connections between a voxel and the rest of the brain (Martuzzi et al. 2011). Connections were computed from the matrix of bivariate correlation coefficients between the BOLD time series from each pair of voxels, estimated using a singular value decomposition (SVD) of the z-score normalized BOLD signal (subject-level SVD) with 64 components, separately for each subject and condition (Whitfield-Gabrieli and Nieto-Castanon 2012). LCOR maps characterizing local coherence at each voxel were estimated as the weighted average of all short-range connections between a voxel and a 25 mm FWHM Gaussian neighbourhood area (Deshpande et al. 2009). Short-range connections were computed from the matrix of bivariate correlation coefficients between the BOLD timeseries from each pair of voxels, estimated using a singular value decomposition of the z-score normalized BOLD signal (subject-level SVD) with 64 components separately for each subject and condition (Whitfield-Gabrieli and Nieto-Castanon 2012). fALFF maps characterizing low-frequency BOLD signal variability at each voxel were estimated as the ratio between the root mean square (RMS) of the BOLD signal after denoising and band-pass filtering between 0.008 Hz and 0.09 Hz, divided by the same measure computed before band-pass filtering (Zou et al. 2008). We aggregated the voxel-based metrics to predefined Regions of Interest (ROIs) using the default atlas in the CONN Toolbox. It combines the FSL Harvard–Oxford atlas at cortical and subcortical areas and the AAL atlas at cerebellar regions, yielding 132 ROIs. The 132 regions were used as inputs for the statistical analysis.

### Laterality index

The laterality index (LI) is a quantitative measure to assess the degree of hemispheric asymmetry in brain function, connectivity, or activation patterns (Brumer et al. 2020; Chlebus et al. 2007). It provides a numerical representation of hemispheric lateralisation of different measured values. Equation 1 shows the general formula for computing the laterality index:

1 $$\:LI=\frac{L-R}{L+R}$$

Laterality Index calculation where L represents the value of a specific metric obtained from a region of interest (ROI) in the Left hemisphere, and R represents the value of the same metric obtained from its homologous ROI in the Right hemisphere.

A LI value of 0 indicates no lateralisation, meaning balanced activity of the two hemispheres in the given brain function. The magnitude of the LI reflects the predominance of lateralisation. Positive vs. negative values indicate lateralisation of the given function to the left or to the right hemisphere, respectively.

In our study, while acknowledging the documented limitations of the LI formula when applied directly to fMRI data e.g., sensitivity to thresholding, arbitrary region-of-interest definitions, and potential for misinterpretation of true hemispheric dominance in certain contexts (Brumer et al. 2020) we applied this formula to prespecified and calculated metrics taking these considerations into account. Specifically, we calculated the LI for both the voxel-based (IC, LCOR, fALFF) and graph-based (GE, LE, BC, APL, CC, degree and cost) rs-fMRI metrics for the previously mentioned 132 regions in all participants.

For each of the 132 ROIs, a single value was derived for each metric as described in Sect. 2.5 and 2.6. When calculating the LI for a given metric, ‘L’ corresponded to the calculated value of that metric for an ROI in the left hemisphere, and ‘R’ corresponded to the value of the same metric for its anatomically homologous ROI in the right hemisphere. This approach allowed us to quantify the hemispheric asymmetry of these derived measures, providing insights into the lateralisation of specific functional and connectivity patterns after initial data processing and metric computation.

### Statistical analysis

To assess the comparability of the ADNI and Local cohorts, demographic and clinical characteristics were compared. Specifically, Independent Samples t-tests were utilized to compare age, education level, and MMSE scores between the two cohorts. A Chi-square test was applied to compare the distribution of sex across the cohorts.

For the investigation of laterality in the normal control group, statistical analyses were conducted using Python programming language and relevant libraries, including statsmodels, SciPy, and NumPy. Multiple paired t-tests were conducted individually to examine the differences between the left and right hemispheres for each metric. For each metric, the null hypothesis was that there were no significant differences in the mean values between the left and right hemispheres. Bonferroni correction was applied to adjust for multiple comparisons, as separate t-tests were performed for each metric. The significance threshold for each t-test was adjusted by dividing the alpha level (typically 0.05) by the total number of brain regions (*n* = 132). Cohen’s d was applied to calculate the effect size for each t-test. Cohen’s d not only represents the effect size, but also reflects on the lateralisation, i.e. a positive value represents leftward lateralisation while a negative one represents a rightward lateralisation. Additionally, one-sample t-tests were performed to assess if the LI values significantly deviate from 0, indicating a significant lateralisation effect. Bonferroni corrections and Cohen’s d calculations were also applied.

## Results

### Demographics

The age of the participants in the Local and the ADNI groups was not significantly different. In both groups, the number of female participants was slightly higher than that of males; however, the difference was not significant, and the effect size indicated a negligible effect. The mean duration of education was similar in the two groups. The Mini Mental State Examination test demonstrated significant differences between the two cohorts, however both groups remained in the cognitively normal range (Salis et al. 2023). Demographic data are presented in Table 1.

**Table 1:** Demographic data of the participants of the two independent cohorts

Parameter	Local dataset (*n*: 102)	ADNI dataset (*n*: 86)	*p*-value	Effect size
Age in years (mean, SD)	66 ± 7.5	68 ± 4.3	0.059	0.29
Sex (% of females)	68	60	0.32	0.1
Education in years (median, IQ)	16.15 ± 3.0	16.6 ± 2.3	0.26	0.16
MMSE (mean, SD)	28.58 ± 1.1	29.1 ± 1.0	0.006	0.51

*MMSE *Mini-mental state examination test

### Graph metric-based approach

We found only a limited number of regions where the graph metrics, after Bonferroni correction, showed significant differences in resting-state connectivity between the left and right hemispheres in both the Local and ADNI datasets. In the Local dataset, significant differences were observed in the supracalcarine cortex, where the metric APL had a p-value of less than 0.001 and an effect size of 0.29. Additionally, the Cerebellum 6 showed significant differences for multiple metrics: GE (*p* < 0.001, effect size 0.40), Cost and Degree (*p* < 0.001, effect size 0.47), and APL (*p* < 0.001, effect size − 0.33). For the ADNI dataset, a significant difference was identified in the juxtapositional lobule cortex for the Cost and Degree metric, with a p-value of less than 0.001 and an effect size of −0.31.

However, without Bonferroni correction, multiple regions showed significant left-right differences of connectivity both in the Local (Supplementary Table 1.) and in the ADNI dataset (Supplementary Table 2.). We chose to include these uncorrected results to provide a more comprehensive view of our findings, as they reveal patterns that are consistent with the voxel-based metrics after correction. This inclusion allows for a more nuanced interpretation of the data, acknowledging that the strict Bonferroni correction may overly penalize the exploratory nature of our study. Additionally, considering recent findings on the limited reproducibility of graph metrics (Telesford et al. 2010), these uncorrected results may still provide valuable insights into lateralisation that voxel-based metrics could validate.

When we compared the data from the Local and ADNI datasets without applying the Bonferroni correction, we found that several regions showing a significant difference between the left and right hemispheres overlapped in the two cohorts (Supplementary Table 3). However, there were no overlapping regions when the Bonferroni-corrected data were analysed.

### Graph-based laterality index

Similarly, to the regular graph-based metrics, we found only two regions where the LI, after Bonferroni correction, showed significant differences between the left and right hemispheres in the Local and ADNI datasets. In the Local dataset, significant differences were observed in the central opercular cortex for the Cost and Degree metric (*p* < 0.001, effect size 0.503). The cerebellum 6 also exhibited differences for multiple metrics, including GE (*p* < 0.001, effect size 0.591), Cost and Degree (*p* < 0.001, effect size 0.631), and APL (*p* < 0.001, effect size − 0.506). In the ADNI dataset, a significant difference was found in the juxtapositional lobule cortex for the Cost and Degree metric, with a p-value of less than 0.001 and an effect size of −0.313.

While multiple regions showed significant left-right differences of connectivity both in the local (Supplementary Table 4.) and in the ADNI dataset (Supplementary Table 5).

**Fig. 1:**
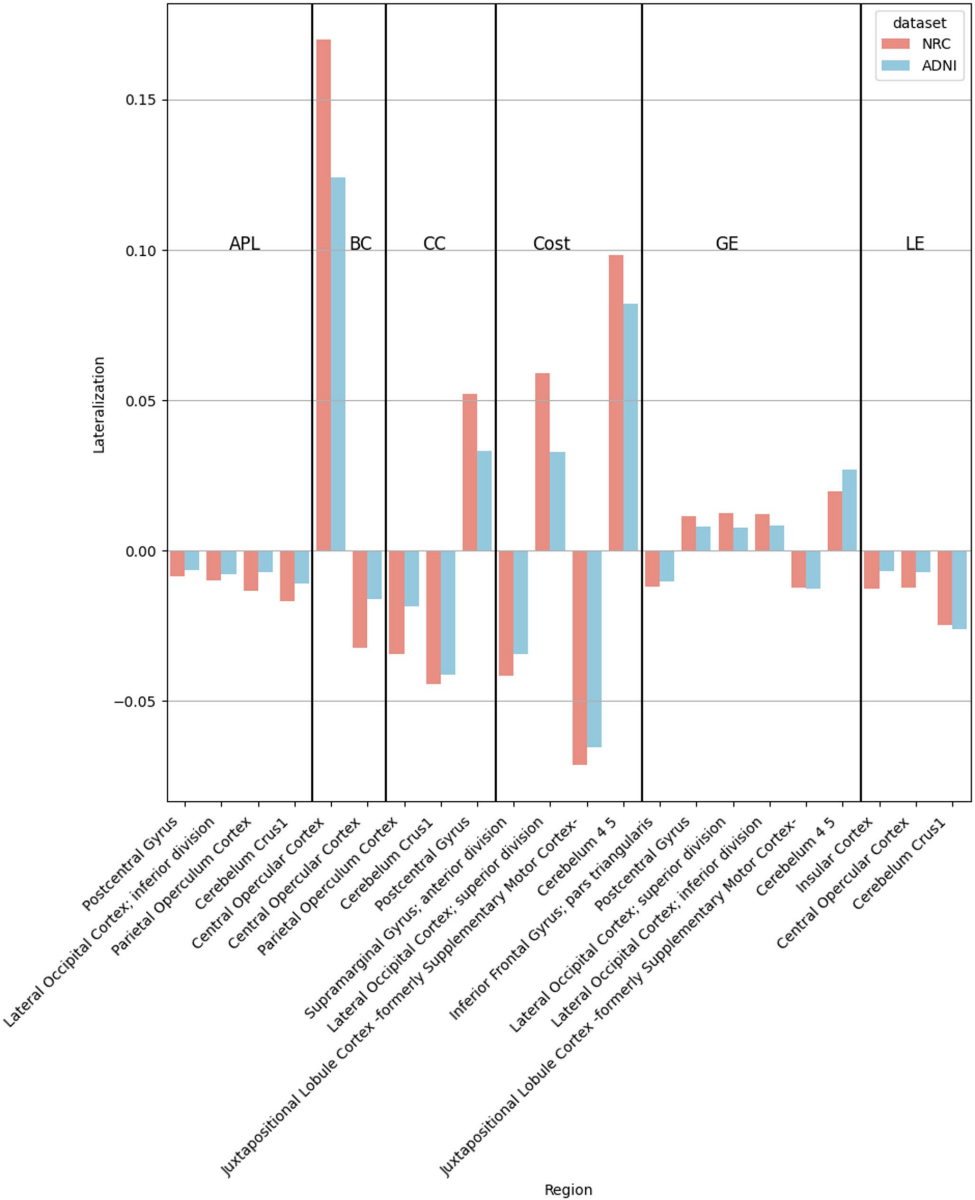
Lateralisation in Graph-Based Metrics, Direction of lateralisation in various brain regions derived from graph-metrics for the Local and ADNI datasets without Bonferroni correction. On the y axis positive values indicate left-sided, while negative values indicate right-sided lateralisation of connectivity functions. Blue colour: Local dataset; Red colour: ADNI dataset. *APL* Average path length, *BC* Betweenness centrality, *CC* Clustering coefficient, *LE* Local efficiency, *GE* Global efficiency

A bar plot of the LI in the two datasets can be seen in Fig. 1. to visualize the direction and size of lateralisation. As one of the most important results of the analysis, we found that in those regions where there was lateralisation in the resting state connectivity using the graph-based metrics, the direction of the lateralisation was the same in all regions in the two datasets (see Fig. 1.) However, there was a difference in the preponderance of the lateralisation. Regions shown in Fig. 1. are without Bonferroni correction, since there were no overlapping regions with correction.

### Voxel-based metrics approach

In the Local dataset, regions showing significant differences between the two hemispheres in voxel-based metrics, after Bonferroni correction, are listed in Table 2. The analysis revealed a leftward lateralisation in the Superior Frontal Gyrus, Inferior Frontal Gyrus (pars triangularis and pars opercularis), and the anterior division of the Superior Temporal Gyrus. These regions had significant p-values (*p* < 0.001) and positive effect sizes, indicating stronger connectivity in the left hemisphere. The Inferior Frontal Gyrus, pars triangularis, exhibited one of the largest effect sizes (LCOR: 0.60, fALFF: 0.66), underscoring its prominent role in the observed asymmetry. The Cerebellum Crus2 and Cerebellum 10 regions showed substantial leftward lateralisation (Cerebellum Crus2 LCOR: 0.65, IC: 0.55, fALFF: 1.38; Cerebellum 10 LCOR: 0.57, IC: 1.04, fALFF: 1.71), with the largest effect size observed in the fALFF metric for Cerebellum 10.

**Table 2:** Regions demonstrating significant left/right differences of connectivity in voxel-based metrics after bonferroni correction in the local dataset

Region	Metric	*P*-Values	Effect Sizes	Lateralisation Direction
Superior Frontal Gyrus	LCOR	*p* < 0.001	0,25	Left
Inferior Frontal Gyrus, pars triangularis	LCOR	*p* < 0.001	0,60	Left
Inferior Frontal Gyrus, pars opercularis	LCOR	*p* < 0.001	0,40	Left
Superior Temporal Gyrus, anterior division	LCOR	*p* < 0.001	0,34	Left
Supramarginal Gyrus, posterior division	LCOR	*p* < 0.001	−0,46	Right
Juxtapositional Lobule Cortex	LCOR	*p* < 0.001	−0,18	Right
Paracingulate Gyrus	LCOR	*p* < 0.001	0,17	Left
Temporal Fusiform Cortex, posterior division	LCOR	*p* < 0.001	−0,27	Right
Planum Temporale	LCOR	*p* < 0.001	−0,33	Right
Supracalcarine Cortex	LCOR	*p* < 0.001	−0,20	Right
Cerebellum Crus2	LCOR	*p* < 0.001	0,65	Left
Cerebellum 6	LCOR	*p* < 0.001	0,19	Left
Cerebellum 8	LCOR	*p* < 0.001	0,20	Left
Cerebellum 10	LCOR	*p* < 0.001	0,57	Left
Superior Frontal Gyrus	IC	*p* < 0.001	0,14	Left
Middle Frontal Gyrus	IC	*p* < 0.001	0,21	Left
Inferior Frontal Gyrus, pars triangularis	IC	*p* < 0.001	0,27	Left
Middle Temporal Gyrus, posterior division	IC	*p* < 0.001	0,32	Left
Lateral Occipital Cortex, superior division	IC	*p* < 0.001	0,18	Left
Juxtapositional Lobule Cortex	IC	*p* < 0.001	−0,17	Right
Occipital Pole	IC	*p* < 0.001	0,18	Left
Cerebellum Crus2	IC	*p* < 0.001	0,55	Left
Cerebellum 4 5	IC	*p* < 0.001	0,15	Left
Cerebellum 6	IC	*p* < 0.001	0,19	Left
Cerebellum 7b	IC	*p* < 0.001	−0,27	Right
Cerebellum 8	IC	*p* < 0.001	0,21	Left
Cerebellum 9	IC	*p* < 0.001	−0,18	Right
Cerebellum 10	IC	*p* < 0.001	1,04	Left
Frontal Pole	fALFF	*p* < 0.001	−0,13	Right
Superior Frontal Gyrus	fALFF	*p* < 0.001	0,33	Left
Middle Frontal Gyrus	fALFF	*p* < 0.001	0,26	Left
Inferior Frontal Gyrus, pars triangularis	fALFF	*p* < 0.001	0,66	Left
Inferior Frontal Gyrus, pars opercularis	fALFF	*p* < 0.001	0,50	Left
Superior Temporal Gyrus, posterior division	fALFF	*p* < 0.001	0,39	Left
Inferior Temporal Gyrus, posterior division	fALFF	*p* < 0.001	0,47	Left
Paracingulate Gyrus	fALFF	*p* < 0.001	0,18	Left
Frontal Orbital Cortex	fALFF	*p* < 0.001	0,36	Left
Frontal Operculum Cortex	fALFF	*p* < 0.001	0,49	Left
Cerebellum Crus2	fALFF	*p* < 0.001	1,38	Left
Cerebellum 6	fALFF	*p* < 0.001	0,38	Left
Cerebellum 7b	fALFF	*p* < 0.001	−0,62	Right
Cerebellum 10	fALFF	*p* < 0.001	1,71	Left

*IC* Intrinsic connectivity, *LCOR* Local correlation, *fALFF* fractional Amplitude of Low Frequency Fluctuations

Regions showing significant differences between the two hemispheres in voxel-based metrics after Bonferroni correction for the ADNI dataset are displayed in Table 3.

**Table 3:** Regions demonstrating significant left/right differences of connectivity in voxel-based metrics after bonferroni correction in the ADNI dataset

Region	Metric	*P*-Values	Effect Sizes	Lateralisation Direction
Superior Frontal Gyrus	LCOR	*p* < 0.001	0,16	Left
Inferior Frontal Gyrus, pars triangularis	LCOR	*p* < 0.001	0,63	Left
Inferior Temporal Gyrus, posterior division	LCOR	*p* < 0.001	0,40	Left
Supramarginal Gyrus, posterior division	LCOR	*p* < 0.001	−0,54	Right
Lateral Occipital Cortex, inferior division	LCOR	*p* < 0.001	−0,34	Right
Juxtapositional Lobule Cortex	LCOR	*p* < 0.001	−0,16	Right
Supracalcarine Cortex	LCOR	*p* < 0.001	−0,55	Right
Cerebellum Crus2	LCOR	*p* < 0.001	0,54	Left
Cerebellum 10	LCOR	*p* < 0.001	0,77	Left
Inferior Temporal Gyrus, posterior division	IC	*p* < 0.001	0,18	Left
Cerebellum Crus2	IC	*p* < 0.001	0,41	Left
Cerebellum 7b	IC	*p* < 0.001	−0,30	Right
Cerebellum 10	IC	*p* < 0.001	0,71	Left
Frontal Pole	fALFF	*p* < 0.001	−0,21	Right
Lateral Occipital Cortex, superior division	fALFF	*p* < 0.001	−0,16	Right
Lateral Occipital Cortex, inferior division	fALFF	*p* < 0.001	−0,25	Right
Frontal Orbital Cortex	fALFF	*p* < 0.001	0,32	Left
Supracalcarine Cortex	fALFF	*p* < 0.001	−0,23	Right
Cerebellum Crus1	fALFF	*p* < 0.001	0,58	Left
Cerebellum Crus2	fALFF	*p* < 0.001	1,46	Left
Cerebellum 6	fALFF	*p* < 0.001	0,23	Left
Cerebellum 7b	fALFF	*p* < 0.001	−0,98	Right
Cerebellum 10	fALFF	*p* < 0.001	1,83	Left

*IC* Intrinsic Connectivity, *LCOR* Local Correlation, *fALFF* fractional Amplitude of Low Frequency Fluctuations

The Superior Frontal Gyrus shows a leftward lateralisation with a modest effect size of 0.16, suggesting stronger local coherence in the left hemisphere. The Inferior Frontal Gyrus, pars triangularis, a region associated with language and cognitive control, shows one of the most substantial leftward lateralisation with an effect size of 0.63. Several regions demonstrate a rightward lateralisation, such as the Supramarginal Gyrus, posterior division, and the Supracalcarine Cortex, with negative effect sizes of −0.54 and − 0.55, respectively. The cerebellum also displays significant lateralisation. Cerebellum Crus2 and Cerebellum 10 exhibit leftward lateralisation with effect sizes of 0.54 and 0.77, respectively, in the LCOR metric. Additionally, the Cerebellum 10 region shows a notable leftward lateralisation in the IC metric with an effect size of 0.71. Cerebellar regions also demonstrate large effect sizes in the fALFF metric. These effect sizes reveal a strong dominance of the right hemisphere among these cerebellar regions. fALFF also indicates that the Frontal Pole has a rightward lateralisation with a negative effect size of −0.21. In contrast, the Frontal Orbital Cortex shows a leftward bias with an effect size of 0.32, suggesting variability in the resting-state activity across different brain regions.

In the voxel-based analysis, similarly to the graph-based results, we also found regions that showed significant left/right difference of connectivity both in the Local and the ADNI datasets. These overlapping regions and the relevant metrics are shown in Table 4.

**Table 4:** Regions which showed significant left/right difference of connectivity in voxel-based analysis both in the local and the ADNI dataset

Region	Metric 1	Metric 2	Metric 3
Superior Frontal Gyrus	LCOR		
Inferior Frontal Gyrus, pars triangularis	LCOR		
Supramarginal Gyrus, posterior division	LCOR		
Juxtapositional Lobule Cortex	LCOR		
Supracalcarine Cortex	LCOR		
Cerebellum Crus2	LCOR	IC	fALFF
Cerebellum 10	LCOR	IC	fALFF
Cerebellum 7b		IC	fALFF
Frontal Pole			fALFF
Frontal Orbital Cortex			fALFF
Cerebellum 6			fALFF

*IC* Intrinsic Connectivity, *LCOR* Local Correlation, *fALFF* fractional Amplitude of Low Frequency Fluctuations

### Voxel-based laterality index

When calculating the LI for the voxel-based metrics, several regions significantly differed from 0 in both the ADNI and the Local dataset, with moderate-large effect sizes. Tables 5 and 6 show which measurement methods resulted in significant lateralisation together with the p-values and the effect size values for the Local and the ADNI dataset, respectively. In the Local dataset (Table 5) the Superior Frontal Gyrus and the Inferior Frontal Gyrus (pars triangularis and pars opercularis) exhibit strong leftward lateralisation, with effect sizes of 0.52, 0.58, and 0.43, respectively. The anterior divisions of the Superior and Middle Temporal Gyri similarly show a leftward bias with effect sizes of 0.44 and 0.36. The Supramarginal Gyrus’s posterior division, the Planum Temporale, and the Supracalcarine Cortex are regions that exhibit a rightward lateralisation with effect sizes of −0.59, −0.50, and − 0.41, respectively. The Cerebellum Crus2 displays a profound leftward lateralisation in both LCOR and IC metrics, with effect sizes of 1.06 and 1.22. Cerebellum 10 also shows a strong leftward bias with effect sizes of 0.67 in LCOR and an even more pronounced 1.53 in IC. The fALFF, the Frontal Pole shows a notable rightward lateralisation with an effect size of −0.46. However, the Superior Frontal Gyrus, Middle Frontal Gyrus, and the Inferior Frontal Gyrus (pars triangularis) exhibit significant leftward lateralisation with robust effect sizes of 0.95, 0.56, and 0.81, respectively. The cerebellar regions stand out in the fALFF metric, with Cerebellum Crus2 and Cerebellum 10 having the highest effect sizes of 2.18 and 2.46.

**Table 5:** Regions where the voxel-based laterality index was significantly different from zero after bonferroni correction in the local dataset

Region	Metric	*P*-Values	Effect Sizes	Lateralisation Direction
Superior Frontal Gyrus	LCOR	*p* < 0.0001	0,52	Left
Inferior Frontal Gyrus, pars triangularis	LCOR	*p* < 0.0001	0,58	Left
Inferior Frontal Gyrus, pars opercularis	LCOR	*p* < 0.0001	0,43	Left
Superior Temporal Gyrus, anterior division	LCOR	*p* < 0.0001	0,44	Left
Middle Temporal Gyrus, anterior division	LCOR	*p* < 0.0001	0,36	Left
Supramarginal Gyrus, posterior division	LCOR	*p* < 0.0001	−0,59	Right
Paracingulate Gyrus	LCOR	*p* < 0.0001	0,41	Left
Lingual Gyrus	LCOR	*p* < 0.0001	0,36	Left
Temporal Fusiform Cortex, posterior division	LCOR	*p* < 0.0001	−0,37	Right
Planum Temporale	LCOR	*p* < 0.0001	−0,50	Right
Supracalcarine Cortex	LCOR	*p* < 0.0001	−0,41	Right
Cerebellum Crus2	LCOR	*p* < 0.0001	1,06	Left
Cerebellum 6	LCOR	*p* < 0.0001	0,39	Left
Cerebellum 8	LCOR	*p* < 0.0001	0,45	Left
Cerebellum 10	LCOR	*p* < 0.0001	0,67	Left
Superior Frontal Gyrus	IC	*p* < 0.0001	0,36	Left
Middle Frontal Gyrus	IC	*p* < 0.0001	0,42	Left
Inferior Frontal Gyrus, pars triangularis	IC	*p* < 0.0001	0,42	Left
Superior Temporal Gyrus, posterior division	IC	*p* < 0.0001	0,37	Left
Middle Temporal Gyrus, posterior division	IC	*p* < 0.0001	0,44	Left
Lateral Occipital Cortex, superior division	IC	*p* < 0.0001	0,45	Left
Juxtapositional Lobule Cortex	IC	*p* < 0.0001	−0,36	Right
Occipital Pole	IC	*p* < 0.0001	0,43	Left
Cerebellum Crus2	IC	*p* < 0.0001	1,22	Left
Cerebellum 4 5	IC	*p* < 0.0001	0,39	Left
Cerebellum 6	IC	*p* < 0.0001	0,56	Left
Cerebellum 7b	IC	*p* < 0.0001	−0,38	Right
Cerebellum 8	IC	*p* < 0.0001	0,42	Left
Cerebellum 9	IC	*p* < 0.0001	−0,47	Right
Cerebellum 10	IC	*p* < 0.0001	1,53	Left
Frontal Pole	fALFF	*p* < 0.0001	−0,46	Right
Superior Frontal Gyrus	fALFF	*p* < 0.0001	0,95	Left
Middle Frontal Gyrus	fALFF	*p* < 0.0001	0,56	Left
Inferior Frontal Gyrus, pars triangularis	fALFF	*p* < 0.0001	0,81	Left
Inferior Frontal Gyrus, pars opercularis	fALFF	*p* < 0.0001	0,62	Left
Superior Temporal Gyrus, posterior division	fALFF	*p* < 0.0001	0,45	Left
Inferior Temporal Gyrus, posterior division	fALFF	*p* < 0.0001	0,50	Left
Paracingulate Gyrus	fALFF	*p* < 0.0001	0,44	Left
Frontal Orbital Cortex	fALFF	*p* < 0.0001	0,60	Left
Frontal Operculum Cortex	fALFF	*p* < 0.0001	0,59	Left
Cerebellum Crus2	fALFF	*p* < 0.0001	2,18	Left
Cerebellum 6	fALFF	*p* < 0.0001	0,72	Left
Cerebellum 7b	fALFF	*p* < 0.0001	−0,62	Right
Cerebellum 10	fALFF	*p* < 0.0001	2,46	Left

*IC* Intrinsic connectivity, *LCOR* Local correlation, *fALFF* fractional Amplitude of Low Frequency Fluctuations

In the ADNI cohort (Table 6) the Superior Frontal Gyrus and the Inferior Frontal Gyrus (pars triangularis) show strong left hemisphere lateralisation with effect sizes of 0.50 and 0.69, respectively, when measured with LCOR. The posterior division of the Inferior Temporal Gyrus also exhibits leftward asymmetry with an LCOR effect size of 0.52. In contrast, the Supramarginal Gyrus (posterior division) and several other regions, including the Lateral Occipital Cortex (inferior division), Juxtapositional Lobule Cortex, and the Supracalcarine Cortex, show a preference for the right hemisphere, with the Supracalcarine Cortex showing a particularly high effect size of −1.28 in LCOR. Cerebellum Crus2 and Cerebellum 10 display marked leftward lateralisation in both LCOR and IC metrics with effect sizes of 0.91 and 1.22 for LCOR, and 1.02 and 1.63 for IC. The fALFF metric shows a notable rightward bias in the Frontal Pole (effect size of −0.72), Lateral Occipital Cortex (both superior and inferior divisions with effect sizes of −0.41 and − 0.43), and the Supracalcarine Cortex (effect size of −0.43). However, the cerebellar regions, particularly Cerebellum Crus1, Crus2, and Cerebellum 10, show a strong leftward bias in fALFF with effect sizes of 0.75, 2.05, and 2.48, respectively. Cerebellum 7b shows a pronounced rightward lateralisation with an fALFF effect size of −1.51.

**Table 6:** Regions where the voxel-based laterality index was significantly different from zero after bonferroni correction in the ADNI dataset

Region	Metric	*P*-Values	Effect Sizes	Latralisation direction
Superior Frontal Gyrus	LCOR	*p* < 0.001	0,50	Left
Inferior Frontal Gyrus, pars triangularis	LCOR	*p* < 0.001	0,69	Left
Inferior Temporal Gyrus, posterior division	LCOR	*p* < 0.001	0,52	Left
Supramarginal Gyrus, posterior division	LCOR	*p* < 0.001	−0,63	Right
Lateral Occipital Cortex, inferior division	LCOR	*p* < 0.001	−0,46	Right
Juxtapositional Lobule Cortex	LCOR	*p* < 0.001	−0,49	Right
Supracalcarine Cortex	LCOR	*p* < 0.001	−1,28	Right
Cerebellum Crus2	LCOR	*p* < 0.001	0,91	Left
Cerebellum 10	LCOR	*p* < 0.001	1,22	Left
Inferior Temporal Gyrus, posterior division	IC	*p* < 0.001	0,45	Left
Frontal Orbital Cortex	IC	*p* < 0.001	0,40	Left
Cerebellum Crus2	IC	*p* < 0.001	1,02	Left
Cerebellum 6	IC	*p* < 0.001	0,41	Left
Cerebellum 7b	IC	*p* < 0.001	−0,60	Right
Cerebellum 10	IC	*p* < 0.001	1,63	Left
Frontal Pole	fALFF	*p* < 0.001	−0,72	Right
Lateral Occipital Cortex, superior division	fALFF	*p* < 0.001	−0,41	Right
Lateral Occipital Cortex, inferior division	fALFF	*p* < 0.001	−0,43	Right
Frontal Orbital Cortex	fALFF	*p* < 0.001	0,49	Left
Supracalcarine Cortex	fALFF	*p* < 0.001	−0,43	Right
Cerebellum Crus1	fALFF	*p* < 0.001	0,75	Left
Cerebellum Crus2	fALFF	*p* < 0.001	2,05	Left
Cerebellum 6	fALFF	*p* < 0.001	0,43	Left
Cerebellum 7b	fALFF	*p* < 0.001	−1,51	Right
Cerebellum 10	fALFF	*p* < 0.001	2,48	Left

*IC* Intrinsic connectivity, *LCOR* Local correlation, *fALFF* fractional Amplitude of Low Frequency Fluctuations

**Fig. 2:**
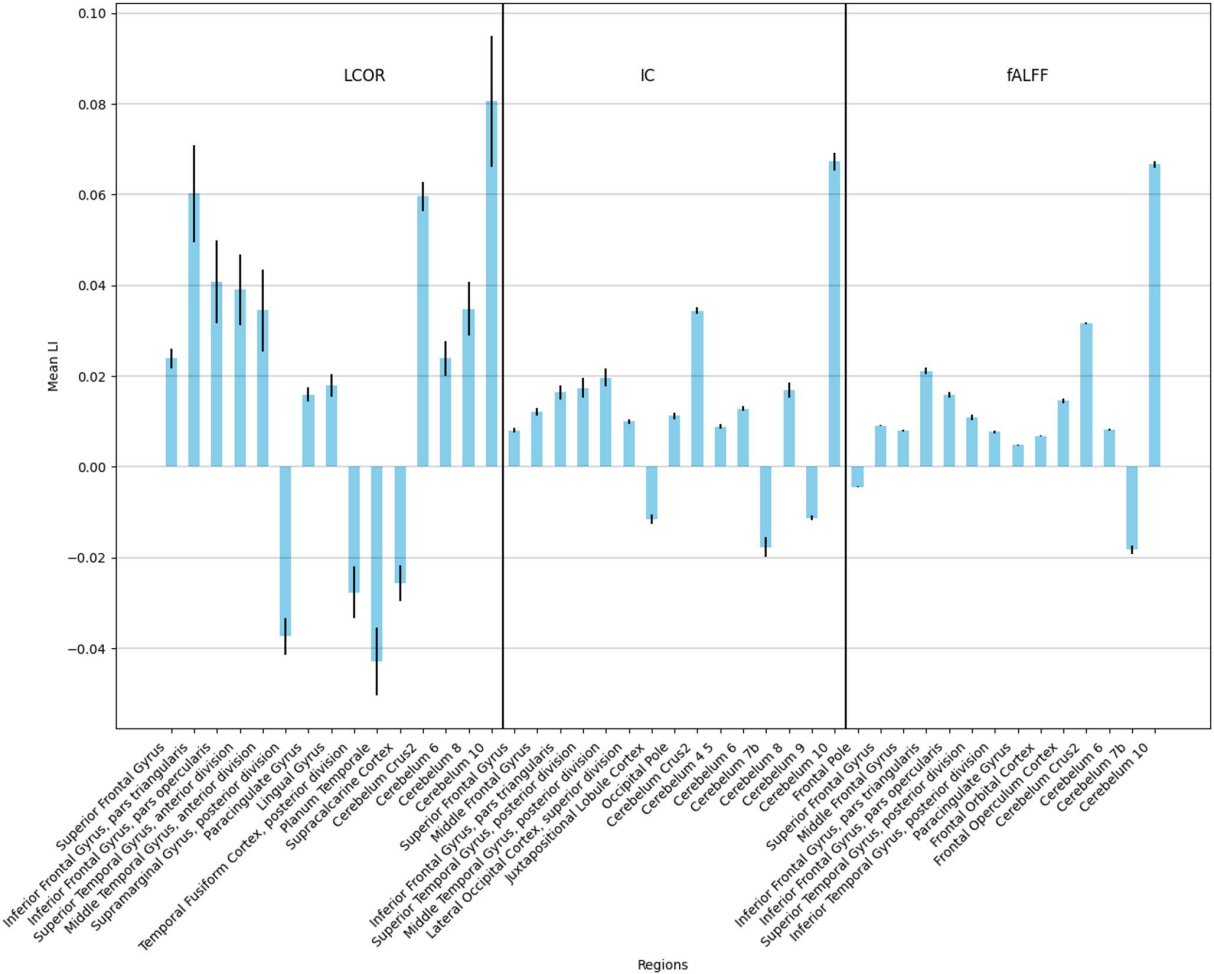
Voxel-Based Laterality Index – Local Dataset, Bar plot of the voxel-based mean laterality index values of the regions that are significantly different from 0 in the Local dataset after Bonferroni correction. Error bars indicate the variance in the data. Positive values on the y axis indicate left-sided lateralisation, while negative values show right-sided lateralisation. *IC* Intrinsic connectivity, *LCOR* Local correlation, *fALFF* fractional Amplitude of Low Frequency Fluctuations

**Fig. 3:**
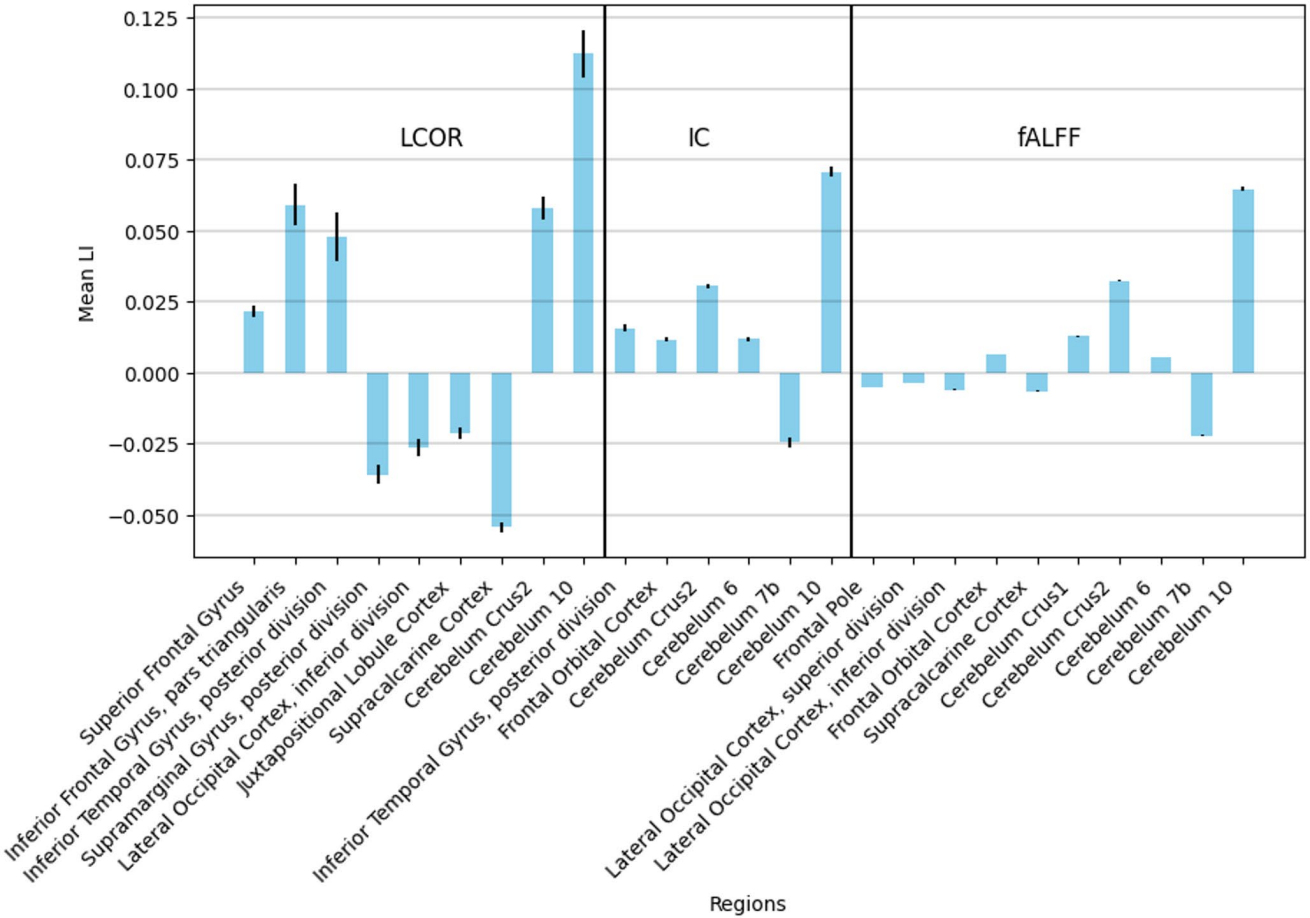
Voxel-Based Laterality Index – ADNI Dataset, Bar plot of the voxel-based mean laterality index values of the regions that are significantly different from 0 in the ADNI dataset after Bonferroni correction. Error bars indicate the variance in the data. Positive values on the y axis indicate left-sided lateralisation, while negative values show right-sided lateralisation. *IC* Intrinsic connectivity, *LCOR* Local correlation, *fALFF* fractional Amplitude of Low Frequency Fluctuations

The mean laterality index values of the various brain regions that are significantly different from 0 after Bonferroni correction both in the Local and in the ADNI datasets are presented in Figs. 2 and 3, respectively.

**Fig. 4:**
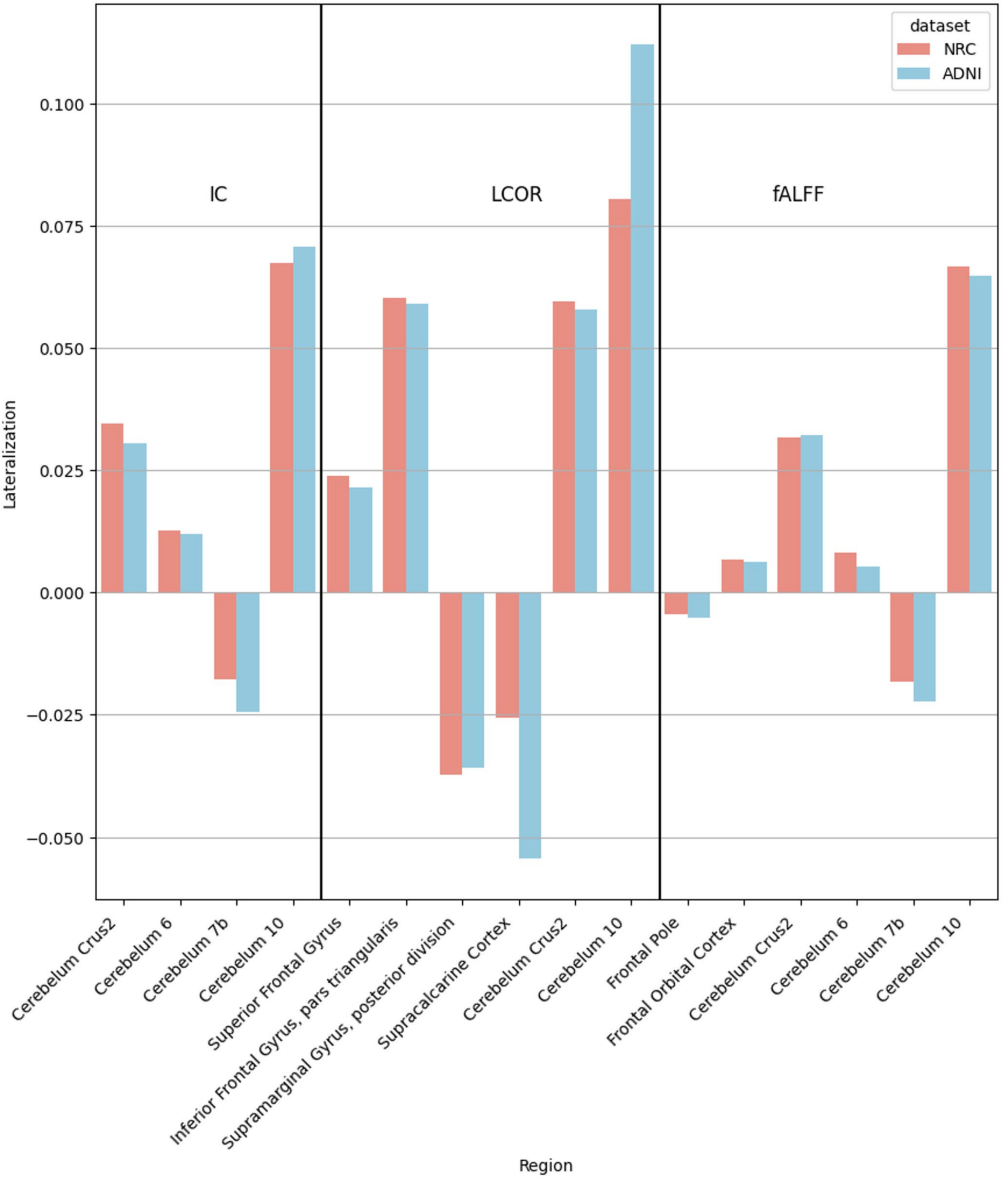
Comparison of Laterality Index Across Datasets, Voxel-based laterality bar plot for the Local and ADNI datasets after Bonferroni correction. On the y axis positive values indicate left-sided, while negative values indicate right-sided lateralisation of connectivity functions. Blue colour: Local dataset; Red colour: ADNI dataset. *IC* Intrinsic connectivity, *LCOR* Local correlation, *fALFF* fractional Amplitude of Low Frequency Fluctuations

A bar plot of the LI in the two datasets is shown in Fig. 4. Similarly, to the results of the graph-based analysis, we found that in all brain regions where there was a significant difference of connectivity between the two hemispheres the direction of the lateralisation was the same in the Local and the ADNI datasets (see Fig. 4). Results shown in Fig. 4. contain regions after Bonferroni correction.

### Regional overlap of connectivity lateralisation derived from the voxel-based laterality metrics in the independent datasets

To see how concordant the direction of the lateralisation of rs-fMRI connectivity results are in the various brain areas in the two independent datasets we analysed which regions showed the same direction of hemispheric voxel-based LI after Bonferroni correction. The direction of LI was the same in brain regions listed in Table 7. It is important to note that there were four regions where the direction of the lateralisation was the same in the Local and the ADNI cohorts, but in different metrics (Supracalcarine Cortex, Lateral Occipital Cortex, Juxtapositional Lobule Cortex, Inferior Temporal Gyrus, posterior division).

**Table 7:** Regions that show the same direction of lateralisation both in the local and the ADNI dataset, the metrics where the overlap was found and the direction of lateralisation

Region	Metric	Lateralisation direction
Cerebellum 10	LCOR, IC, fALFF	Left
Cerebellum Crus2	LCOR, IC, fALFF	Left
Cerebellum 6	IC, fALFF	Left
Cerebellum 7b	IC fALFF	Right
Frontal Orbital Cortex	fALFF	Left
Frontal Pole	fALFF	Right
Inferior Frontal Gyrus, pars triangularis	LCOR	Left
Superior Frontal Gyrus	LCOR	Left
Supramarginal Gyrus, posterior division	LCOR	Left
Supracalcarine Cortex	None	Right
Lateral Occipital Cortex	None	Left
Juxtapositional Lobule Cortex	None	Right
Inferior Temporal Gyrus, posterior division	None	Left

*IC* Intrinsic connectivity, *LCOR* Local correlation, *fALFF* fractional Amplitude of Low Frequency Fluctuations

**Fig. 5:**
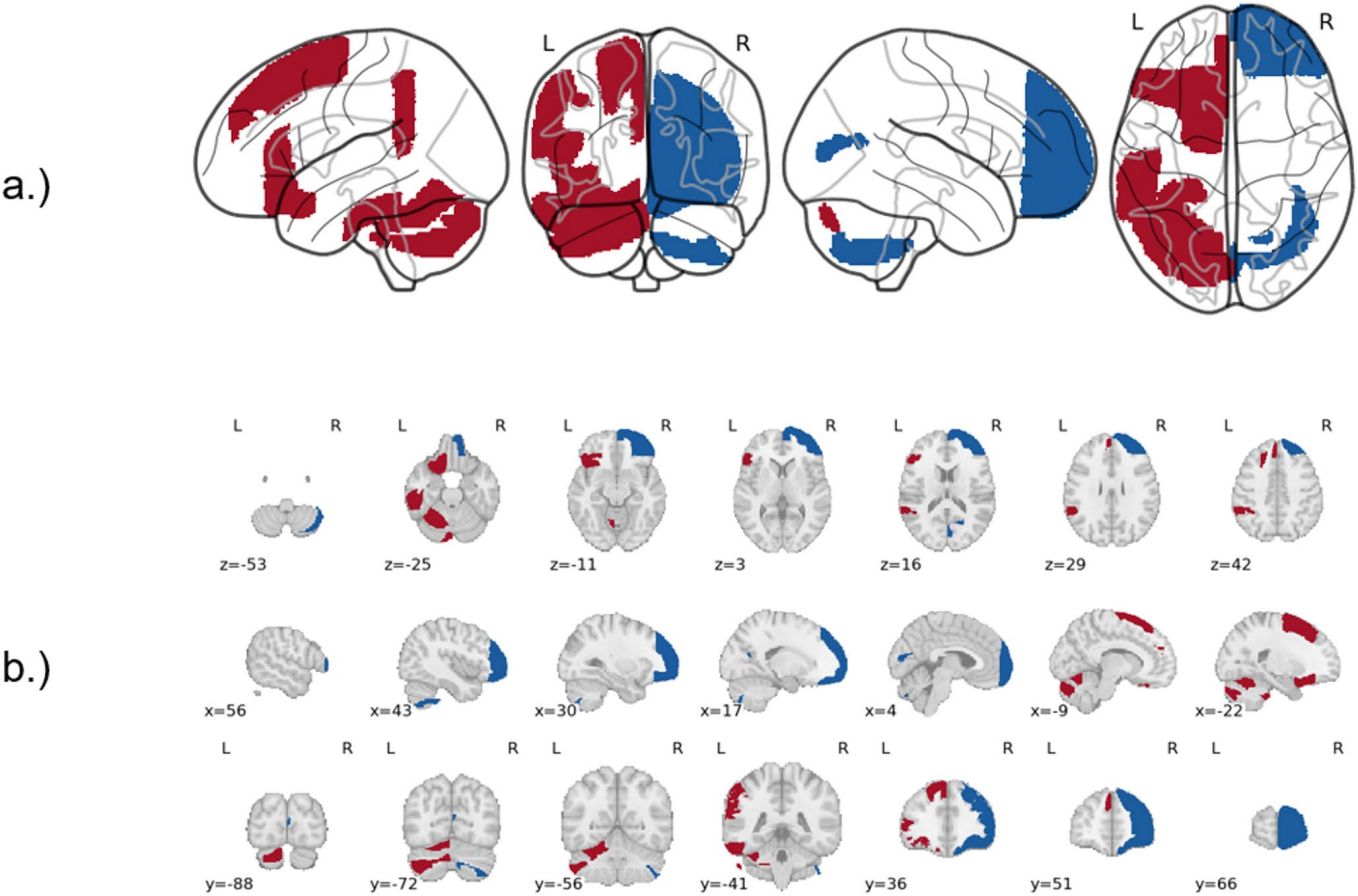
Brain Regions with Consistent Lateralisation **a** Glass brain of regions showing the same side of hemispheric lateralisation derived from the voxel-based laterality index rs-fMRI data Red colour depicts regions with left hemispheric lateralisation (Cerebellum 10, Cerebellum 6, Cerebellum Crus2, Frontal Orbital Cortex, Inferior Frontal Gyrus, pars triangularis, Inferior Temporal Gyrus, posterior division, Lateral Occipital Cortex, Superior Frontal Gyrus, Supramarginal Gyrus, posterior division) Blue colour depicts regions with right hemispheric lateralisation (Cerebellum 7b, Frontal Pole, Juxtapositional Lobule Cortex, Supracalcarine Cortex) **b** Showing the same results on a mosaic figure across brain slices

Figure 5. shows these regions in a glass and mosaic brain.

## Discussion

In this study, we employed fMRI analysis to investigate lateralisation of resting-state network connectivity in the healthy human brain using a multifaceted analytical approach, which included seven graph-theoretical measures, three voxel-based metrics, and the calculation of the laterality index for the voxel-based metrics. We conducted our study using two distinct, independent fMRI datasets: one national (*n* = 102) and one international (*n* = 86). The concordance of the results from statistical analyses of data originating from independent cohorts confirms the reproducibility of the findings and highlights their importance. The demographic data (age, sex, handedness) of the investigated cohorts were not significantly different, making the information derived from them comparable. MMSE scores showed a significant difference (*p* = 0.006) however both cohorts MMSE scores were in cognitively normal range (> 26) (Salis et al. 2023).

Even after correction for multiple statistical comparisons, many brain regions demonstrated significant resting-state functional network connectivity lateralisation with modest and moderately large effect sizes in both datasets. As one of the main outcomes of our study, the results suggest a consistent hemispheric preponderance in rs-fMRI connectivity across the two databases, even when different analytical methods are employed. More specifically, if there was a significant lateralisation in the resting-state connectivity measures in a given brain region in the two datasets, their side (left or right) was always the same.

Given the consistency of the results across multiple analysis methods, we identified 13 brain regions out of 132 in both datasets, with powerful lateralisation features. These regions are the following: Cerebellum 10, Cerebellum 6, Cerebellum 7b, Cerebellum Crus2, Frontal Orbital Cortex, Frontal Pole, Inferior Frontal Gyrus (pars triangularis), Inferior Temporal Gyrus (posterior division), Juxtapositional Lobule Cortex, Lateral Occipital Cortex (superior division), Superior Frontal Gyrus, Supracalcarine Cortex, and Supramarginal Gyrus (posterior division). 

Overall, the frontal and temporal cortical areas, as well as the cerebellum, exhibited a dominant left-sided lateralisation pattern of connectivity, whereas the parietal regions demonstrated a right-sided lateralisation. 

In contrast to the everyday use of VMHC to assess hemispheric symmetry (Zuo et al. 2010; Agcaoglu et al. 2015), our study employs a novel approach by directly comparing metrics between corresponding regions across the hemispheres. Analysis of graph metrics revealed similar lateralisation directions in the two datasets; however, this was only observed for a limited number of regions. Lateralisation of connectivity measures becomes less pronounced when Bonferroni correction for multiple comparisons is applied, suggesting that the observed asymmetries detected with graph metrics may be statistically fragile. Recent results suggested that graph metrics have limited reproducibility (Telesford et al. 2010), while a more granular examination using voxel-based metrics (Hayasaka and Laurienti 2010) reveals regions with consistently reproducible lateralisation. Therefore, the diminishing statistical significance could be attributed to the reproducibility issues with graph metrics. Additionally, it is important to note that graph-theoretical and voxel-based approaches inherently capture different aspects of brain organization: graph metrics summarize network-level properties and integration, while voxel-based metrics are more sensitive to local or regional effects. As a result, some divergence in the sets of identified regions is expected, reflecting the complementary strengths and limitations of each method. Interestingly, when examining uncorrected graph metric results the lateralisation observed in voxel-based metrics aligns with our graph metrics observations of asymmetry in local efficiency, average path length, and clustering coefficient. We opted to include the results without Bonferroni correction to provide a more comprehensive and nuanced interpretation of our findings. This decision is rooted in the exploratory nature of our study and the observation that the regions identified as significant in the uncorrected graph metrics align well with those found in the corrected voxel-based metrics. By presenting these uncorrected results, we aim to highlight potential patterns and asymmetries that might be biologically relevant but are not detected under the stringent Bonferroni correction. These converging lines of evidence underscore the robustness of lateralisation phenomena across different analytical methods. The consistence of the LI values in the two independent datasets that we used in the current study further supports the presence of asymmetry of cerebral networks and confirms our observations gained from graph-based and voxel-based metrics.

Contrasting with previous works that predominantly focused on the lateralisation of connectivity of brain networks—either structural or functional—we investigated the connectivity of individual brain regions. A landmark study on 346 healthy individuals analysing functional brain networks demonstrated increased connectivity of the left-sided frontal regions related to motor and language control, while more pronounced right-sided connectivity was found in parietal regions involved in visuospatial navigation (Caeyenberghs and Leemans 2014). Consistent with these findings, our study also identifies left-hemispheric dominance in frontal and temporal regions and right-sided dominance in parietal areas regarding resting-state network functions. Most of the previously published reports analysed the connectivity of brain areas as physiological units of large-scale brain networks (see comprehensive review by Fink, and Marshall 2007; and Hervé et al. 2013).

However, the clinical relevance of lateralisation extends beyond physiological function, as it may offer critical insights into the pathophysiology of various neurological conditions. Information about the lateralisation of various brain functions in healthy humans could help us to understand the development and progression of various diseases that are known to present with a left or right hemispheric predominance. In our investigation, regions such as the cerebellum, frontal orbital cortex, frontal pole, inferior frontal gyrus, inferior temporal gyrus, lateral occipital cortex, and supramarginal gyrus demonstrated substantial lateralisation of resting state connectivity in the healthy brain. These cortical areas parallel the regions frequently affected by neuro-psychiatric diseases (like schizophrenia or epilepsy), suggesting a possible link between pre-existing connectivity differences between the two hemispheres and disease manifestation (Lopes et al. 2017; Guardiola-Ripoll and Fatjó-Vilas 2023; Ahmad et al. 2023). Interestingly, the language-dominant hemisphere shows vulnerability in multiple dementia-related conditions characterized by a spreading disease pathology. Histopathological studies in Parkinson’s disease demonstrated more severe alpha-synuclein deposition in the dominant hemisphere corresponding to motor symptom dominance (Uchihara 2017). Alzheimer’s studies also showed that cortical neurodegeneration occurs earlier and faster in the left hemisphere (Janke et al. 2001; Toga and Thompson 2003), in line with the pronounced vulnerability of the left hemisphere to amyloid deposition (Frings et al. 2015). Moreover, as the disease progresses, the left-oriented neuronal connectivity becomes depleted, and the frontotemporal networks show a rightward topological asymmetry (Daianu et al. 2013; Yang et al. 2017). The asymmetric distribution pattern of TDP-43 protein (more in the language-dominant hemisphere) has been implicated in patients with frontotemporal dementia (Irwin et al. 2017) and primary progressive aphasia as well (Kim et al. 2016).

It is well documented, that pathological hyperexcitability is more pronounced in the dominant hemisphere in epilepsy. Two early studies examined ~ 1300 EEG reports each and found that interictal epileptiform discharges (IED) were two times more common over the left hemisphere (Dean et al. 1997; Doherty et al. 2002). In case of bilateral discharges, left-sided spikes were identified more frequently in 80% of the patients (Dean et al. 1997). The analysis of the spatial distribution of IEDs on > 30.000 EEG recordings obtained from > 24.000 patients reported a significantly higher proportion of left-sided discharges with increased age-related lateralisation tendency (Loddenkemper et al. 2007). Interestingly, the left dominance was detected not only for IEDs (79% on the left) but for all focal EEG abnormalities (77%) in the study of Gatzonis et al. (2002). A recent observation systematically evaluating 99 patients with focal seizures found that seizure onset zones were 2 times more common in the left hemisphere, and left-sided seizure-onset zone occurrence was associated with higher seizure frequency, earlier disease onset, and worse response for antiseizure medications (Varoglu 2024). Other studies also reported worse clinical outcomes in seizures originating from the left hemisphere and a higher frequency of associated depression (Mendez et al. 1986). A growing body of evidence suggests that epileptiform activity/hyperexcitability also shows a left-sided tendency in Alzheimer’s disease. Subclinical epileptiform activity (SEA: spikes detected on the EEG of patients without overt seizures) shows 2 times more frequent occurrence over the left hemisphere in patients with Alzheimer’s disease (Kamondi et al. 2024). Importantly, the left-sided unilateral or the left-dominant bilateral occurrence of SEA was associated with a tendency for faster disease progression (Horvath et al. 2021). This phenomenon is barely understood; however, recent observations have highlighted that regions with amyloid accumulation highly overlap with epileptogenic foci (Lam et al. 2023, 2024). Thus, the left-predominant asymmetric pattern of hyperexcitability might be the result of the asymmetric pathologic protein deposition in Alzheimer’s disease.

Based on the above-referenced articles, lateralisation plays a central role in the pathophysiology of neurological diseases, such as neurodegenerative disorders and epilepsy. Various hypotheses explain the predisposition of the dominant hemisphere for the primary onset of pathology, including the relation to handedness, epigenetic differences across the hemispheres, asymmetry in cytoarchitecture and neurochemistry, and lateralisation of the pathologic triggers (for a comprehensive review, see Lubben et al. (2021). However, it is barely understood why any pathological process affecting the left side (the language-dominant hemisphere in 85% of the population) is associated with poorer prognosis and clinical outcomes.

A possible explanation is that the dominant hemisphere provides more optimal conditions for the faster spread of the pathology, due to its more widespread long-distance connections within the involved neural networks. Multiple studies showed that the spreading of pathological amyloid in Alzheimer’s disease is related to the functional network organization (Powell et al. 2018; Pereira et al. 2019; Fornari et al. 2020). Notably, the spreading pattern respects the preformed functional connectome, despite deteriorating white matter integrity (Powell et al. 2018). Furthermore, epilepsy studies demonstrated that epileptic discharges generate aberrant network organization and long-distance remodelling in the functional networks related initially to the seizure onset zone (Kramer and Cash 2012; Fahoum et al. 2012; Yaffe et al. 2015). It is intriguing to postulate that an ongoing pathology in epilepsy would generate more severe and long-lasting changes in the neural system (parallel to worse clinical status and prognosis) if the onset zone has more widespread structural and functional connections to multiple brain areas. According to the current literature (Holmes et al. 2010; García-Cabrero et al. 2013; Scheltens et al. 2021), neurological disorders affecting the frontotemporal cortices (e.g., Alzheimer’s disease, frontotemporal dementia, epilepsy) show a higher tendency for dominant hemispheric onset, and this type of asymmetry is associated with more severe clinical representation. Based on our analysis, the resting-state connectivity functions in the temporal regions consistently exhibited left-sided lateralisation across all assessed metrics in healthy individuals, regardless of the dataset studied. The consistency of the direction of lateralisation corroborates this left-sided dominance, reinforcing the hypothesis that pre-existing asymmetries may influence the hemispherical onset and progression of neurological disease.

One limitation of this study is its exclusive focus on a single age cohort, which restricts the generalizability of our findings across the lifespan. A longitudinal approach, capturing data from individuals of various ages, could yield valuable insights into the evolution of lateralisation of connectivity functions and their compensatory mechanisms. Additionally, while rigorous multiple comparison correction methods were applied to mitigate type I errors, the inherent variability and potential biases associated with statistical thresholds and methods must be considered. The adoption of alternative analytical strategies in future research may serve to validate or refine our findings. Another limitation arises from our study’s focus on individual brain regions, which may inadvertently diminish the significance of inter-regional networks and their lateralisation patterns. A complementary network-based analysis could provide a better perspective on the regional approach employed here, potentially revealing a more integrated view of brain asymmetry. An additional point to consider is the exclusive focus on healthy individuals. While understanding healthy circumstances represents a key motivation to understand disease physiology, conclusions may remain speculative, and clinical implications are only indirect. Further studies are needed to compare the health characteristics with disease populations (e.g., patients with focal epilepsy, neurodegenerative diseases, or gliomas). Communication of healthy samples and analysis methods is the first step, representing the significant value of the current study.

Concluding the future directions, our comprehensive analysis of brain lateralisation of resting-state fMRI connectivity data, using multiple measures and independent datasets, has identified key hemispheric and cerebellar regions that exhibit significant hemispheric lateralisation. These findings enhance our understanding of the complex relations between brain asymmetry and might facilitate further research on the pathophysiology of various neurological diseases. These studies could potentially help to understand the development and progression of various symptoms in patients living with neurologic and psychiatric disorders. As a potential clinical implication, lateralisation of disease pathology in neurological and psychiatric disorders may be implemented as a prognostic and/or phenotyping marker. Lateralisation indices might assist in making personalized clinical decisions by identifying individuals with clinically aggressive phenotypes. Moving forward, the integration of longitudinal and network-based approaches, along with the inclusion of diverse age groups and patient populations, will be essential for building a more comprehensive picture of brain function lateralisation throughout the human lifespan.

## Supplementary Information

Below is the link to the electronic supplementary material.

Supplementary Material 1

Supplementary Material 2

Supplementary Material 3

Supplementary Material 4

Supplementary Material 5

## Data Availability

The entire dataset is available upon reasonable request sent to the corresponding author.
