# Supplementary material for: Hemispheric asymmetries in resting-state connectivity: insights from healthy controls and implications for neurological disorders
Source: Brain Struct Funct. 2025 Nov 10;230(9):174. doi: 10.1007/s00429-025-03039-8 (PMC12602572; doi:10.1007/s00429-025-03039-8)
Supplement: Supplementary file 1 — Supplementary Material 1 [file 429_2025_3039_MOESM1_ESM.docx]

| **Region** | **Metric** | **P-Values** | **Effect Sizes** | **Lateralisation Direction** |
| --- | --- | --- | --- | --- |
| Insular Cortex | LE | 0,031 | -0,20 | Right |
| Middle Frontal Gyrus | LE | 0,012 | 0,33 | Left |
| Middle Frontal Gyrus | CC | 0,021 | 0,28 | Left |
| Inferior Frontal Gyrus; pars triangularis | GE | 0,024 | -0,24 | Right |
| Inferior Frontal Gyrus; pars triangularis | Cost and Degree | 0,037 | -0,25 | Right |
| Inferior Frontal Gyrus; pars triangularis | APL | 0,027 | 0,23 | Left |
| Inferior Frontal Gyrus; pars opercularis | LE | 0,003 | 0,36 | Left |
| Inferior Frontal Gyrus; pars opercularis | BC | 0,023 | -0,32 | Right |
| Inferior Frontal Gyrus; pars opercularis | CC | 0,002 | 0,41 | Left |
| Superior Temporal Gyrus; anterior division | BC | 0,049 | 0,29 | Left |
| Middle Temporal Gyrus; posterior division | GE | 0,040 | -0,22 | Right |
| Middle Temporal Gyrus; posterior division | APL | 0,038 | 0,21 | Left |
| Postcentral Gyrus | GE | 0,011 | 0,18 | Left |
| Postcentral Gyrus | LE | 0,020 | -0,25 | Right |
| Postcentral Gyrus | Cost and Degree | 0,007 | 0,18 | Left |
| Postcentral Gyrus | APL | 0,034 | -0,15 | Right |
| Postcentral Gyrus | CC | 0,010 | -0,26 | Right |
| Lateral Occipital Cortex; superior division | GE | 0,048 | 0,20 | Left |
| Lateral Occipital Cortex; inferior division | GE | 0,025 | 0,17 | Left |
| Lateral Occipital Cortex; inferior division | APL | 0,020 | -0,20 | Right |
| Juxtapositional Lobule Cortex -formerly Supplementary Motor Cortex- | GE | 0,002 | -0,27 | Right |
| Juxtapositional Lobule Cortex -formerly Supplementary Motor Cortex- | Cost and table | 0,000 | -0,31 | Right |
| Juxtapositional Lobule Cortex -formerly Supplementary Motor Cortex- | APL | 0,013 | 0,19 | Left |
| Paracingulate Gyrus | GE | 0,033 | -0,21 | Right |
| Paracingulate Gyrus | LE | 0,036 | 0,24 | Left |
| Paracingulate Gyrus | BC | 0,010 | -0,37 | Right |
| Paracingulate Gyrus | APL | 0,019 | 0,21 | Left |
| Paracingulate Gyrus | CC | 0,023 | 0,24 | Left |
| Parahippocampal Gyrus; anterior division | Cost and Degree | 0,014 | 0,25 | Left |
| Temporal Fusiform Cortex; posterior division | GE | 0,020 | 0,20 | Left |
| Temporal Fusiform Cortex; posterior division | BC | 0,007 | 0,41 | Left |
| Temporal Fusiform Cortex; posterior division | Cost and Degree | 0,030 | 0,22 | Left |
| Central Opercular Cortex | LE | 0,022 | -0,20 | Right |
| Central Opercular Cortex | BC | 0,001 | 0,42 | Left |
| Central Opercular Cortex | CC | 0,035 | -0,17 | Right |
| Parietal Operculum Cortex | LE | 0,036 | -0,21 | Right |
| Parietal Operculum Cortex | APL | 0,038 | -0,21 | Right |
| Parietal Operculum Cortex | CC | 0,045 | -0,19 | Right |
| Heschl's Gyrus | GE | 0,008 | 0,31 | Left |
| Heschl's Gyrus | Cost and Degree | 0,007 | 0,33 | Left |
| Heschl's Gyrus | APL | 0,011 | -0,28 | Right |
| Cerebellum Crus1 | LE | 0,010 | -0,37 | Right |
| Cerebellum Crus1 | BC | 0,001 | 0,48 | Left |
| Cerebellum Crus1 | APL | 0,048 | -0,14 | Right |
| Cerebellum Crus1 | CC | 0,019 | -0,32 | Right |
| Cerebellum 4 5 | GE | 0,002 | 0,22 | Left |
| Cerebellum 4 5 | BC | 0,004 | 0,41 | Left |
| Cerebellum 4 5 | Cost and Degree | 0,001 | 0,28 | Left |
| Cerebellum 8 | LE | 0,017 | -0,20 | Right |
| Cerebellum 8 | APL | 0,037 | -0,14 | Right |
| Cerebellum 8 | CC | 0,019 | -0,24 | Right |

*Supplementary Table 2. Regions showing significant differences of connectivity between the left and right hemispheres in graph metrics without Bonferroni correction in the ADNI dataset*

Abbreviations: GE: Global Efficiency, APL: Average Path Length, LE: Local Efficiency, CC: Clustering Coefficient, BC: Betweenness Centrality
