# Supplementary material for: Hemispheric asymmetries in resting-state connectivity: insights from healthy controls and implications for neurological disorders
Source: Brain Struct Funct. 2025 Nov 10;230(9):174. doi: 10.1007/s00429-025-03039-8 (PMC12602572; doi:10.1007/s00429-025-03039-8)
Supplement: Supplementary file 2 — Supplementary Material 2 [file 429_2025_3039_MOESM2_ESM.docx]

| **Region** | **Metric 1** | **Metric 2** | **Metric 3** | **Metric 4** | **Metric 5** | **Metric 6** |
| --- | --- | --- | --- | --- | --- | --- |
| Insular Cortex | LE |  |  |  |  |  |
| Inferior Frontal Gyrus; pars triangularis |  | GE | Cost and Degree |  |  |  |
| Postcentral Gyrus |  | GE | Cost and Degree | APL |  |  |
| Lateral Occipital Cortex; superior division |  | GE |  |  |  |  |
| Lateral Occipital Cortex; inferior division |  | GE |  | APL |  |  |
| Juxtapositional Lobule Cortex -formerly Supplementary Motor Cortex |  | GE | Cost and Degree |  |  |  |
| Central Opercular Cortex | LE |  |  |  | CC |  |
| Parietal Operculum Cortex |  |  |  | APL | CC |  |
| Cerebellum Crus1 | LE |  |  | APL | CC | BC |
| Cerebellum 4 5 |  | GE | Cost and Degree |  |  | BC |

*Supplementary Table 3. Regions which showed significant left/right difference of connectivity by graph metrics both in the Local and the ADNI dataset. Data were analysed without Bonferroni correction.*

Abbreviations: GE: Global Efficiency, APL: Average Path Length, LE: Local Efficiency, CC: Clustering Coefficient, BC: Betweenness Centrality
