# Supplementary material for: Hemispheric asymmetries in resting-state connectivity: insights from healthy controls and implications for neurological disorders
Source: Brain Struct Funct. 2025 Nov 10;230(9):174. doi: 10.1007/s00429-025-03039-8 (PMC12602572; doi:10.1007/s00429-025-03039-8)
Supplement: Supplementary file 3 — Supplementary Material 3 [file 429_2025_3039_MOESM3_ESM.docx]

| **Region** | **Metric** | **P-Values** | **Effect Sizes** | **Lateralisation Direction** |
| --- | --- | --- | --- | --- |
| Insular Cortex | LE | 0.027 | -0.245 | Right |
| Middle Frontal Gyrus | LE | 0.015 | 0.270 | Left |
| Middle Frontal Gyrus | CC | 0.028 | 0.242 | Left |
| Inferior Frontal Gyrus; pars triangularis | GE | 0.032 | -0.236 | Right |
| Inferior Frontal Gyrus; pars triangularis | APL | 0.020 | 0.256 | Left |
| Inferior Frontal Gyrus; pars opercularis | GE | 0.049 | -0.217 | Right |
| Inferior Frontal Gyrus; pars opercularis | LE | 0.004 | 0.319 | Left |
| Inferior Frontal Gyrus; pars opercularis | BC | 0.004 | -0.321 | Right |
| Inferior Frontal Gyrus; pars opercularis | Cost and Degree | 0.044 | -0.222 | Right |
| Inferior Frontal Gyrus; pars opercularis | CC | 0.003 | 0.335 | Left |
| Middle Temporal Gyrus; posterior division | GE | 0.041 | -0.225 | Right |
| Middle Temporal Gyrus; posterior division | APL | 0.037 | 0.230 | Left |
| Postcentral Gyrus | GE | 0.012 | 0.279 | Left |
| Postcentral Gyrus | LE | 0.027 | -0.244 | Right |
| Postcentral Gyrus | Cost and Degree | 0.010 | 0.286 | Left |
| Postcentral Gyrus | APL | 0.030 | -0.240 | Right |
| Postcentral Gyrus | CC | 0.019 | -0.259 | Right |
| Supramarginal Gyrus; anterior division | Cost and Degree | 0.050 | -0.216 | Right |
| Supramarginal Gyrus; posterior division | BC | 0.019 | -0.259 | Right |
| Lateral Occipital Cortex; superior division | GE | 0.041 | 0.225 | Left |
| Lateral Occipital Cortex; superior division | Cost and Degree | 0.045 | 0.221 | Left |
| Lateral Occipital Cortex; inferior division | GE | 0.022 | 0.253 | Left |
| Lateral Occipital Cortex; inferior division | APL | 0.021 | -0.254 | Right |
| Juxtapositional Lobule Cortex -formerly Supplementary Motor Cortex- | GE | 0.002 | -0.341 | Right |
| Juxtapositional Lobule Cortex -formerly Supplementary Motor Cortex- | Cost and Degree | 0.000 | -0.424 | Right |
| Juxtapositional Lobule Cortex -formerly Supplementary Motor Cortex- | APL | 0.013 | 0.274 | Left |
| Paracingulate Gyrus | GE | 0.045 | -0.220 | Right |
| Paracingulate Gyrus | LE | 0.035 | 0.233 | Left |
| Paracingulate Gyrus | BC | 0.019 | -0.260 | Right |
| Paracingulate Gyrus | APL | 0.012 | 0.277 | Left |
| Paracingulate Gyrus | CC | 0.019 | 0.260 | Left |
| Temporal Fusiform Cortex; posterior division | BC | 0.007 | 0.298 | Left |
| Temporal Fusiform Cortex; posterior division | Cost and Degree | 0.020 | 0.258 | Left |
| Central Opercular Cortex | LE | 0.022 | -0.253 | Right |
| Central Opercular Cortex | BC | 0.001 | 0.361 | Left |
| Central Opercular Cortex | CC | 0.036 | -0.232 | Right |
| Parietal Operculum Cortex | LE | 0.037 | -0.230 | Right |
| Parietal Operculum Cortex | APL | 0.036 | -0.232 | Right |
| Parietal Operculum Cortex | CC | 0.043 | -0.223 | Right |
| Heschl's Gyrus | GE | 0.007 | 0.300 | Left |
| Heschl's Gyrus | Cost and Degree | 0.006 | 0.309 | Left |
| Heschl's Gyrus | APL | 0.011 | -0.281 | Right |
| Cerebelum Crus1 | LE | 0.016 | -0.268 | Right |
| Cerebelum Crus1 | APL | 0.046 | -0.220 | Right |
| Cerebelum Crus1 | CC | 0.017 | -0.264 | Right |
| Cerebelum 4 5 | GE | 0.036 | 0.231 | Left |
| Cerebelum 4 5 | Cost and Degree | 0.001 | 0.358 | Left |

Supplementary Table 5. Regions where the graph-based Laterality Index was significantly different from zero correction in the ADNI dataset.

Abbreviations: GE: Global Efficiency, APL: Average Path Length, LE: Local Efficiency, CC: Clustering Coefficient, BC: Betweenness Centrality
