# Supplementary material for: Hemispheric asymmetries in resting-state connectivity: insights from healthy controls and implications for neurological disorders
Source: Brain Struct Funct. 2025 Nov 10;230(9):174. doi: 10.1007/s00429-025-03039-8 (PMC12602572; doi:10.1007/s00429-025-03039-8)
Supplement: Supplementary file 4 — Supplementary Material 4 [file 429_2025_3039_MOESM4_ESM.docx]

| **Region** | **Metric** | **P-Values** | **Effect Sizes** | **Lateralisation Direction** |
| --- | --- | --- | --- | --- |
| Insular Cortex | LE | 0.013 | -0.318 | Right |
| Insular Cortex | BC | 0.024 | 0.288 | Left |
| Insular Cortex | CC | 0.010 | -0.328 | Right |
| Superior Frontal Gyrus | Cost and Degree | 0.018 | 0.302 | Left |
| Inferior Frontal Gyrus; pars triangularis | GE | 0.044 | -0.255 | Right |
| Precentral Gyrus | LE | 0.014 | -0.312 | Right |
| Precentral Gyrus | BC | 0.044 | 0.255 | Left |
| Precentral Gyrus | CC | 0.012 | -0.322 | Right |
| Inferior Temporal Gyrus; posterior division | GE | 0.037 | 0.265 | Left |
| Inferior Temporal Gyrus; posterior division | BC | 0.037 | 0.264 | Left |
| Inferior Temporal Gyrus; posterior division | Cost and Degree | 0.002 | 0.399 | Left |
| Postcentral Gyrus | GE | 0.003 | 0.390 | Left |
| Postcentral Gyrus | Cost and Degree | 0.002 | 0.405 | Left |
| Postcentral Gyrus | GE | 0.012 | -0.322 | Right |
| Supramarginal Gyrus; anterior division | LE | 0.002 | -0.409 | Right |
| Supramarginal Gyrus; anterior division | Cost and Degree | 0.041 | -0.258 | Right |
| Supramarginal Gyrus; anterior division | CC | 0.007 | -0.348 | Right |
| Supramarginal Gyrus; posterior division | LE | 0.021 | -0.294 | Right |
| Supramarginal Gyrus; posterior division | CC | 0.020 | -0.297 | Right |
| Angular Gyrus | GE | 0.043 | 0.256 | Left |
| Angular Gyrus | GE | 0.022 | -0.290 | Right |
| Lateral Occipital Cortex; superior division | GE | 0.009 | 0.333 | Left |
| Lateral Occipital Cortex; superior division | Cost and Degree | 0.005 | 0.363 | Left |
| Lateral Occipital Cortex; superior division | GE | 0.035 | -0.267 | Right |
| Lateral Occipital Cortex; inferior division | GE | 0.006 | 0.354 | Left |
| Lateral Occipital Cortex; inferior division | Cost and Degree | 0.007 | 0.345 | Left |
| Lateral Occipital Cortex; inferior division | GE | 0.011 | -0.326 | Right |
| Intracalcarine Cortex | GE | 0.032 | -0.272 | Right |
| Intracalcarine Cortex | Cost and Degree | 0.027 | -0.280 | Right |
| Juxtapositional Lobule Cortex -formerly Supplementary Motor Cortex- | GE | 0.015 | -0.310 | Right |
| Juxtapositional Lobule Cortex -formerly Supplementary Motor Cortex- | Cost and Degree | 0.001 | -0.416 | Right |
| Frontal Orbital Cortex | GE | 0.017 | 0.303 | Left |
| Frontal Orbital Cortex | Cost and Degree | 0.023 | 0.289 | Left |
| Frontal Orbital Cortex | GE | 0.018 | -0.300 | Right |
| Parahippocampal Gyrus; anterior division | LE | 0.030 | -0.276 | Right |
| Central Opercular Cortex | GE | 0.001 | 0.450 | Left |
| Central Opercular Cortex | LE | 0.002 | -0.393 | Right |
| Central Opercular Cortex | BC | 0.001 | 0.420 | Left |
| Central Opercular Cortex | Cost and Degree | 0.000 | 0.503 | Left |
| Central Opercular Cortex | GE | 0.002 | -0.393 | Right |
| Central Opercular Cortex | CC | 0.001 | -0.416 | Right |
| Parietal Operculum Cortex | GE | 0.047 | 0.251 | Left |
| Parietal Operculum Cortex | GE | 0.018 | -0.302 | Right |
| Parietal Operculum Cortex | CC | 0.017 | -0.304 | Right |
| Supracalcarine Cortex | GE | 0.001 | -0.440 | Right |
| Supracalcarine Cortex | Cost and Degree | 0.003 | -0.380 | Right |
| Supracalcarine Cortex | GE | 0.000 | 0.461 | Left |
| Occipital Pole | GE | 0.046 | 0.253 | Left |
| Cerebelum Crus1 | GE | 0.004 | 0.369 | Left |
| Cerebelum Crus1 | LE | 0.005 | -0.359 | Right |
| Cerebelum Crus1 | BC | 0.002 | 0.411 | Left |
| Cerebelum Crus1 | Cost and Degree | 0.025 | 0.285 | Left |
| Cerebelum Crus1 | GE | 0.001 | -0.414 | Right |
| Cerebelum Crus1 | CC | 0.006 | -0.351 | Right |
| Cerebelum 4 5 | GE | 0.008 | 0.337 | Left |
| Cerebelum 4 5 | BC | 0.007 | 0.348 | Left |
| Cerebelum 4 5 | Cost and Degree | 0.004 | 0.373 | Left |
| Cerebelum 4 5 | GE | 0.017 | -0.303 | Right |
| Cerebelum 6 | GE | 0.000 | 0.591 | Left |
| Cerebelum 6 | BC | 0.001 | 0.429 | Left |
| Cerebelum 6 | Cost and Degree | 0.000 | 0.631 | Left |
| Cerebelum 6 | GE | 0.000 | -0.506 | Right |
| Cerebelum 9 | GE | 0.018 | 0.301 | Left |
| Cerebelum 9 | GE | 0.007 | -0.344 | Left |

*Supplementary Table 4. Regions where the graph-based Laterality Index was significantly different from zero correction in the Local dataset.*

Abbreviations: GE: Global Efficiency, APL: Average Path Length, LE: Local Efficiency, CC: Clustering Coefficient, BC: Betweenness Centrality
