# Supplementary material for: Hemispheric asymmetries in resting-state connectivity: insights from healthy controls and implications for neurological disorders
Source: Brain Struct Funct. 2025 Nov 10;230(9):174. doi: 10.1007/s00429-025-03039-8 (PMC12602572; doi:10.1007/s00429-025-03039-8)
Supplement: Supplementary file 5 — Supplementary Material 5 [file 429_2025_3039_MOESM5_ESM.docx]

| **Region** | **Metric** | **P-Values** | **Effect Sizes** | **Lateralisation Direction** |
| --- | --- | --- | --- | --- |
| Insular Cortex | LE | 0,014 | -0,30 | Right |
| Insular Cortex | CC | 0,015 | -0,28 | Right |
| Superior Frontal Gyrus | Cost and Degree | 0,012 | 0,26 | Left |
| Inferior Frontal Gyrus; pars triangularis | GE | 0,040 | -0,30 | Right |
| Inferior Frontal Gyrus; pars triangularis | Cost and Degree | 0,046 | -0,31 | Right |
| Inferior Frontal Gyrus; pars opercularis | Cost and Degree | 0,006 | -0,36 | Right |
| Precentral Gyrus | LE | 0,013 | -0,33 | Right |
| Precentral Gyrus | CC | 0,015 | -0,30 | Right |
| Inferior Temporal Gyrus; posterior division | GE | 0,010 | 0,36 | Left |
| Inferior Temporal Gyrus; posterior division | Cost and Degree | 0,001 | 0,46 | Left |
| Postcentral Gyrus | GE | 0,003 | 0,28 | Left |
| Postcentral Gyrus | Cost and Degree | 0,003 | 0,27 | Left |
| Postcentral Gyrus | APL | 0,009 | -0,25 | Right |
| Supramarginal Gyrus; anterior division | LE | 0,001 | -0,41 | Right |
| Supramarginal Gyrus; anterior division | Cost and Degree | 0,021 | -0,31 | Right |
| Supramarginal Gyrus; anterior division | CC | 0,006 | -0,36 | Right |
| Supramarginal Gyrus; posterior division | LE | 0,022 | -0,26 | Right |
| Supramarginal Gyrus; posterior division | CC | 0,023 | -0,25 | Right |
| Angular Gyrus | GE | 0,048 | 0,26 | Left |
| Angular Gyrus | APL | 0,018 | -0,28 | Right |
| Lateral Occipital Cortex; superior division | GE | 0,011 | 0,32 | Left |
| Lateral Occipital Cortex; superior division | Cost and Degree | 0,006 | 0,37 | Left |
| Lateral Occipital Cortex; superior division | APL | 0,029 | -0,25 | Right |
| Lateral Occipital Cortex; superior division | Cost and Degree | 0,006 | 0,37 | Left |
| Lateral Occipital Cortex; inferior division | GE | 0,006 | 0,25 | Left |
| Lateral Occipital Cortex; inferior division | APL | 0,010 | -0,22 | Right |
| Lateral Occipital Cortex; inferior division | Degree and Degree | 0,007 | 0,25 | Left |
| Intracalcarine Cortex | GE | 0,031 | -0,15 | Right |
| Intracalcarine Cortex | Cost and Degree | 0,026 | -0,17 | Right |
| Juxtapositional Lobule Cortex -formerly Supplementary Motor Cortex- | GE | 0,016 | -0,32 | Right |
| Juxtapositional Lobule Cortex -formerly Supplementary Motor Cortex- | Cost and Degree | 0,002 | -0,42 | Right |
| Frontal Orbital Cortex | GE | 0,021 | 0,29 | Left |
| Frontal Orbital Cortex | APL | 0,017 | -0,28 | Right |
| Parahippocampal Gyrus; anterior division | LE | 0,045 | -0,27 | Right |
| Parahippocampal Gyrus; anterior division | BC | 0,029 | 0,37 | Left |
| Central Opercular Cortex | GE | 0,001 | 0,42 | Left |
| Central Opercular Cortex | LE | 0,002 | -0,34 | Right |
| Central Opercular Cortex | Cost and Degree | 0,001 | 0,38 | Left |
| Central Opercular Cortex | APL | 0,002 | -0,41 | Right |
| Central Opercular Cortex | CC | 0,001 | -0,36 | Right |
| Parietal Operculum Cortex | APL | 0,016 | -0,40 | Right |
| Parietal Operculum Cortex | CC | 0,018 | -0,38 | Right |
| Supracalcarine Cortex | GE | 0,001 | -0,29 | Right |
| Supracalcarine Cortex | Cost and Degree | 0,010 | -0,25 | Right |
| Supracalcarine Cortex | APL | 0,000 | 0,29 | Left |
| Occipital Pole | APL | 0,035 | -0,27 | Right |
| Cerebellum Crus1 | GE | 0,005 | 0,28 | Left |
| Cerebellum Crus1 | LE | 0,003 | -0,49 | Right |
| Cerebellum Crus1 | BC | 0,029 | 0,36 | Left |
| Cerebellum Crus1 | APL | 0,001 | -0,26 | Right |
| Cerebellum Crus1 | CC | 0,002 | -0,44 | Right |
| Cerebellum 4 5 | GE | 0,006 | 0,30 | Left |
| Cerebellum 4 5 | BC | 0,013 | 0,43 | Left |
| Cerebellum 4 5 | Cost and Degree | 0,003 | 0,38 | Left |
| Cerebellum 4 5 | APL | 0,024 | -0,23 | Right |
| Cerebellum 6 | GE | 0,000 | 0,40 | Left |
| Cerebellum 6 | BC | 0,026 | 0,27 | Left |
| Cerebellum 6 | Cost and Degree | 0,000 | 0,47 | Left |
| Cerebellum 6 | APL | 0,000 | -0,33 | Right |
| Cerebellum 9 | GE | 0,022 | 0,19 | Left |
| Cerebellum 9 | APL | 0,011 | -0,25 | Right |

*Supplementary Table 1.* *Regions showing significant differences of connectivity between the left and right hemispheres in graph metrics without Bonferroni correction in the Local database*

Abbreviations: GE: Global Efficiency, APL: Average Path Length, LE: Local Efficiency, CC: Clustering Coefficient, BC: Betweenness Centrality
